# Supplementary material for: H2O2 sulfenylates CHE linking local infection to establishment of systemic acquired resistance
Source: bioRxiv. 2023 Aug 1:2023.07.27.550865. Preprint. [Version 2] doi: 10.1101/2023.07.27.550865 (PMC10402168; doi:10.1101/2023.07.27.550865)
Supplement: Supplement 1 [file NIHPP2023.07.27.550865v2-supplement-1.pdf]

bioRxiv preprint doi: https://doi.org/10.1101/2023.07.27.550865; this version posted August 1, 2023. The copyright holder for this preprint (which was not certified by peer review) is the author/funder, who has granted bioRxiv a license to display the preprint in perpetuity. It is made available under aCC-BY 4.0 International license.

Fig S1

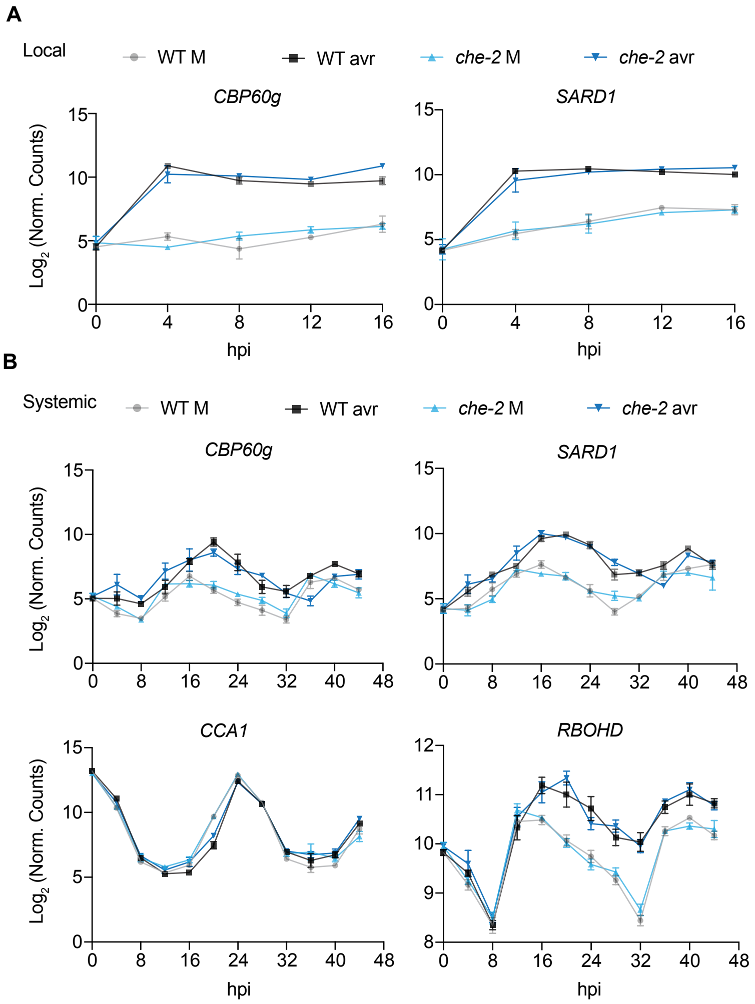

Fig. S1. Normalized read counts from RASL-seq. (**A** and **B**) Normalized transcript read counts in WT and *che-2* plants collected from local (A) and systemic (B) tissues after mock (M; 10 mM $MgCl_2$) or *Psm* ES4326/avrRpt2 (avr; $OD_{600nm} = 0.01$) treatment. Data are means ± SEMs ($n \geq 3$). hpi, hours post infiltration.

bioRxiv preprint doi: https://doi.org/10.1101/2023.07.27.550865; this version posted August 1, 2023. The copyright holder for this preprint (which was not certified by peer review) is the author/funder, who has granted bioRxiv a license to display the preprint in perpetuity. It is made available under aCC-BY 4.0 International license.

Fig S2

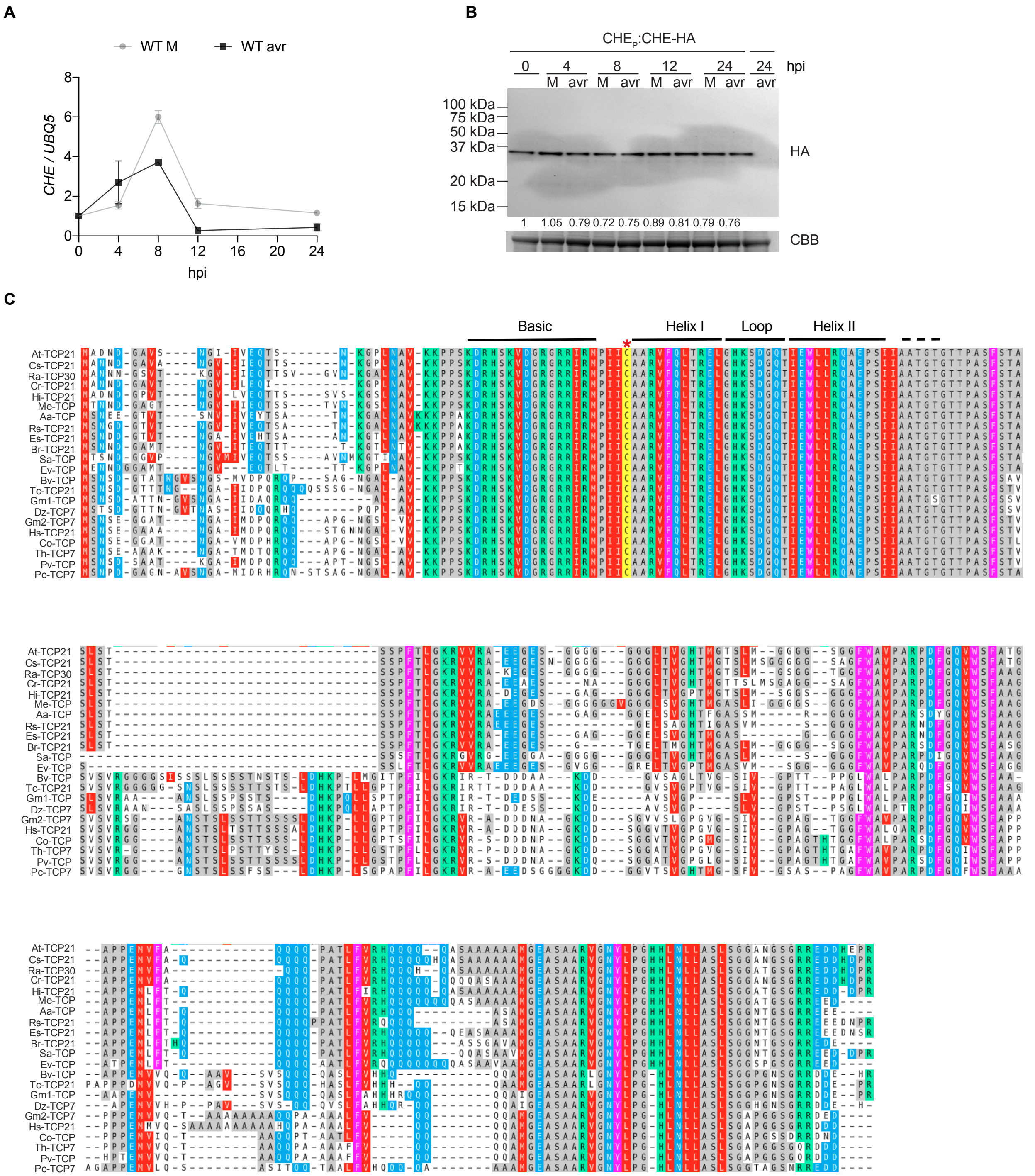

Fig. S2. CHE transcript and protein levels and conservation of the cysteine residue in CHE among plant species. (**A** and **B**) CHE transcript (A) and protein level (B) changes in systemic tissues after local mock (M; 10 mM $MgCl_2$) or *Psm* ES4326/avrRpt2 (avr; OD_{600nm} = 0.01) treatment. hpi, hours post infiltration. Data are means ± SEMs. *n* = 3 for (A). (**C**) Sequence alignment of CHE homologs in plants. The red star indicates the conserved cysteine residue.

bioRxiv preprint doi: https://doi.org/10.1101/2023.07.27.550865; this version posted August 1, 2023. The copyright holder for this preprint (which was not certified by peer review) is the author/funder, who has granted bioRxiv a license to display the preprint in perpetuity. It is made available under aCC-BY 4.0 International license.

Arabidopsis thaliana (*At*), *Camelina sativa* (*Cs*), *Rorippa aquatica* (*Ra*), *Capsella rubella* (*Cr*), *Hirschfeldia incana* (*Hi*), *Microthlaspi erraticum* (*Me*), *Arabis alpina* (*Aa*), *Raphanus sativus* (*Rs*), *Eutrema salsugineum* (*Es*), *Brassica rapa* (*Br*), *Sinapis alba* (*Sa*), *Eruca vesicaria* (*Ev*), *Bauhinia variegate* (*Bv*), *Theobroma cacao* (*Tc*), *Gossypium mustelinum* (*Gm*), *Durio zibethinus* (*Dz*), *Glycine max* (*Gm*), *Hibiscus syriacus* (*Hs*), *Corchorus olitorius* (*Co*), *Tarenaya hassleriana* (*Th*), *Phaseolus vulgaris* (*Pv*), *Prosopis cineraria* (*Pc*).

bioRxiv preprint doi: https://doi.org/10.1101/2023.07.27.550865; this version posted August 1, 2023. The copyright holder for this preprint (which was not certified by peer review) is the author/funder, who has granted bioRxiv a license to display the preprint in perpetuity. It is made available under aCC-BY 4.0 International license.

Fig S3

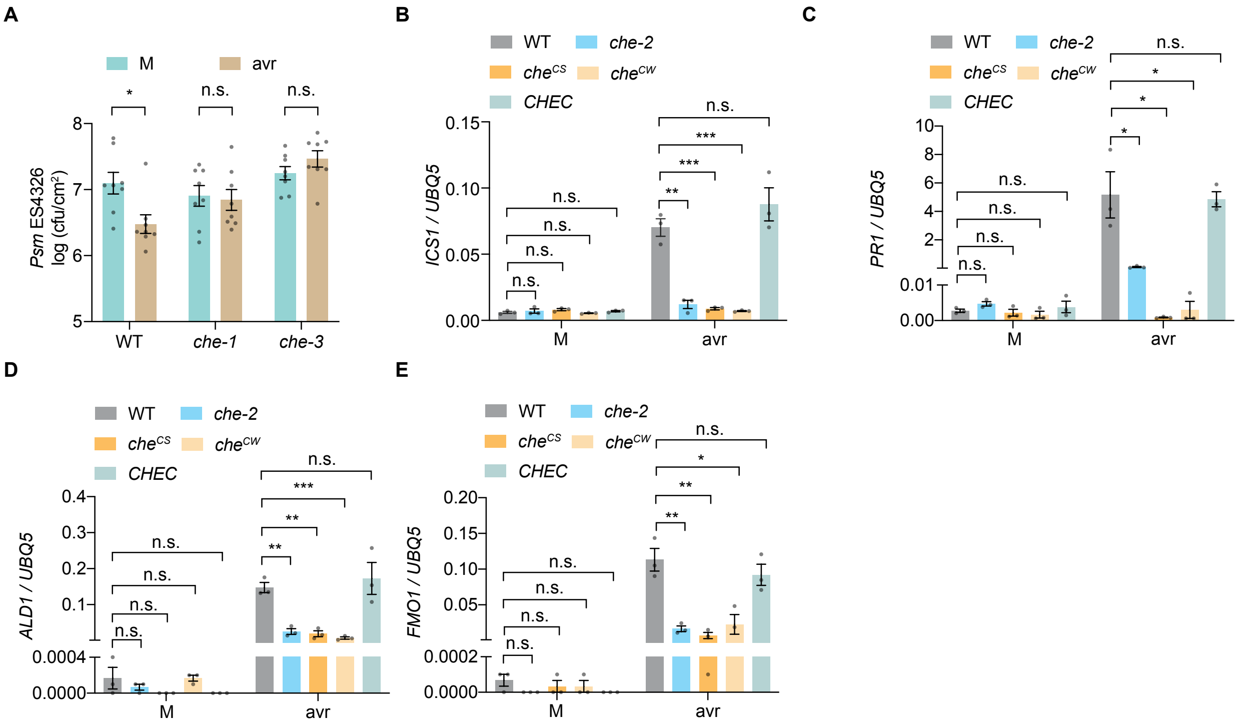

Fig. S3. Bacterial growth and defense-related gene expression in systemic tissues. (**A**) Bacterial growth after pathogen challenge. Plants were infiltrated with mock (M; 10 mM $MgCl_2$) or *Psm* ES4326/avrRpt2 (avr; OD_{600nm} = 0.01) 2 days before infiltration of the systemic tissues with *Psm* ES4326 (OD_{600nm} = 0.001), and bacterial growth was measured 3 days after the second infiltration. Data are means ± SEMs (n = 8). (**B-E**) Transcriptional levels of *ICS1* (B), *ALD1* (C), *FMO1* (D) and *PR1* (E) in systemic tissues after local M or avr treatment. Data are means ± SEMs (n = 3). *CHEC*, *che-2* transformed with the WT *CHE* expressed by its native promoter. *che*^{CS} and *che*^{CW}, transformants expressing the cysteine-to-serine and cysteine-to-tryptophan mutant *che*, respectively. Significant differences were calculated using two-tailed Student's t-tests. ***P < 0.001; **P < 0.01; *P < 0.05; n.s., not significant.

bioRxiv preprint doi: https://doi.org/10.1101/2023.07.27.550865; this version posted August 1, 2023. The copyright holder for this preprint (which was not certified by peer review) is the author/funder, who has granted bioRxiv a license to display the preprint in perpetuity. It is made available under aCC-BY 4.0 International license.

Fig S4

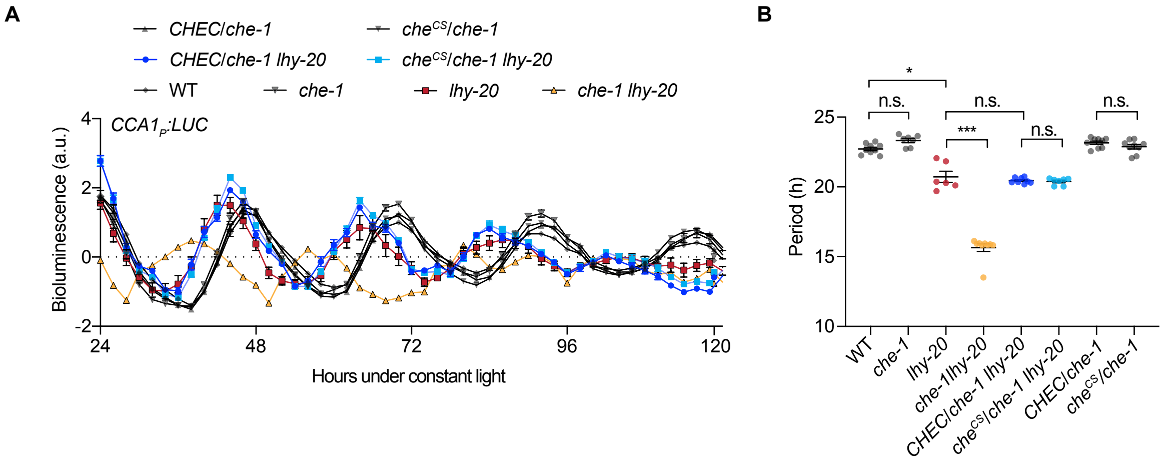

Fig. S4. The cysteine mutant can rescue the phenotype of *che-1* in the regulation of *CCA1*. (**A**) Bioluminescence activity of *CCA1*$_{P}$*:LUC* under constant light condition. Data are means ± SEMs ($n \geq 6$). *CHEC/che-1*, *che-1* transformed with the WT *CHE* expressed by its native promoter. *che*^{CS}, transformants expressing the cysteine-to-serine mutant of CHE. (**B**) Period estimates of *CCA1*$_{P}$*:LUC* activity. Data are means ± SEMs ($n \geq 6$). Significant differences were calculated using two-tailed Student's t-tests. ***$P < 0.001$; **$P < 0.01$; *$P < 0.05$; n.s., not significant.

bioRxiv preprint doi: https://doi.org/10.1101/2023.07.27.550865; this version posted August 1, 2023. The copyright holder for this preprint (which was not certified by peer review) is the author/funder, who has granted bioRxiv a license to display the preprint in perpetuity. It is made available under aCC-BY 4.0 International license.

Fig S5

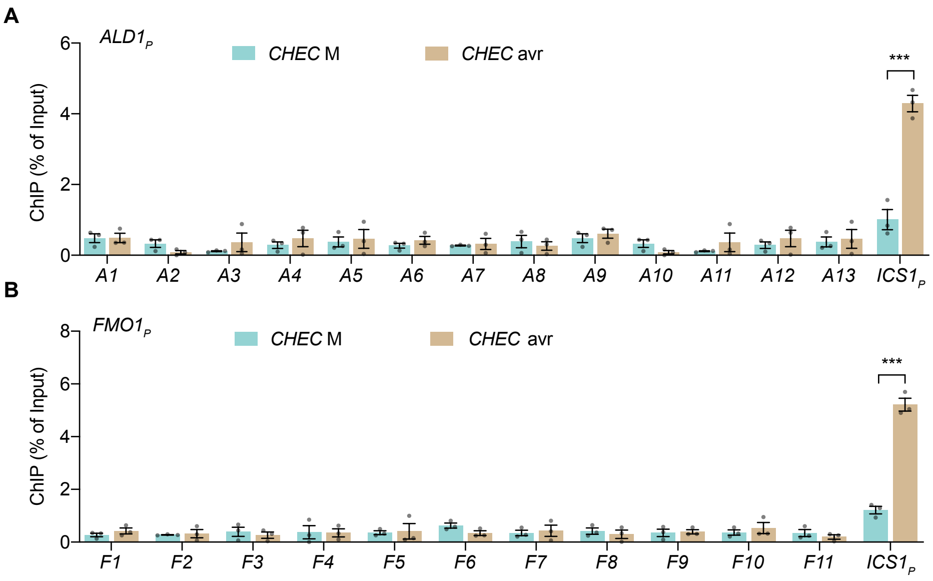

Fig. S5. CHE does not bind to the *ALD1* or *FMO1* promoter. (**A** and **B**) ChIP-qPCR analysis of CHE binding to the *ALD1* promoter (*ALD1P*) (A) and the *FMO1* promoter (*FMO1P*) (B) in systemic tissues 24 hours after local mock (M; 10 mM $MgCl_2$) or *Psm* ES4326/avrRpt2 (avr; OD_{600nm} = 0.01) treatment. *A1* to *A13*, qPCR fragments covering the promoter sequence of *ALD1* (1908 bp upstream ATG to 46 bp downstream ATG); *F1* to *F11*, qPCR fragments covering the promoter sequence of *FMO1* (1583 bp upstream ATG to 53 bp downstream ATG); *ICS1P*, the *ICS1* promoter sequence carrying the TCP-binding site. Data are means ± SEMs (n = 3). Significant differences were calculated using two-tailed Student's t-tests. ***$P < 0.001$.

bioRxiv preprint doi: https://doi.org/10.1101/2023.07.27.550865; this version posted August 1, 2023. The copyright holder for this preprint (which was not certified by peer review) is the author/funder, who has granted bioRxiv a license to display the preprint in perpetuity. It is made available under aCC-BY 4.0 International license.

Fig S6

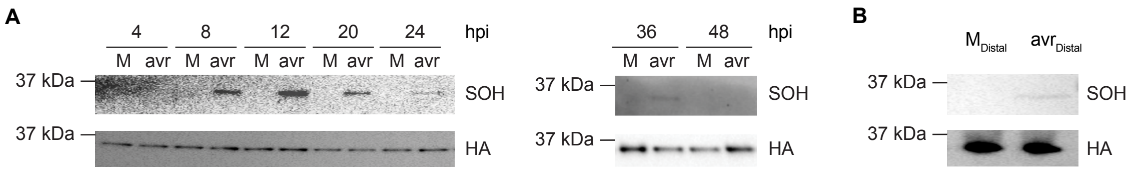

Fig. S6. *In vivo* sulfenylation of CHE. (**A**) Time-course sulfenylation (SOH) of CHE in systemic tissues after local mock (M; 10 mM $MgCl_2$) or *Psm* ES4326/avrRpt2 (avr; OD_{600nm} = 0.01) treatment. hpi, hours post infiltration. (**B**) Sulfenylation of CHE in the distal tissues 24 hours after treatment of the lower leaves with M or avr.

bioRxiv preprint doi: https://doi.org/10.1101/2023.07.27.550865; this version posted August 1, 2023. The copyright holder for this preprint (which was not certified by peer review) is the author/funder, who has granted bioRxiv a license to display the preprint in perpetuity. It is made available under aCC-BY 4.0 International license.

Fig S7

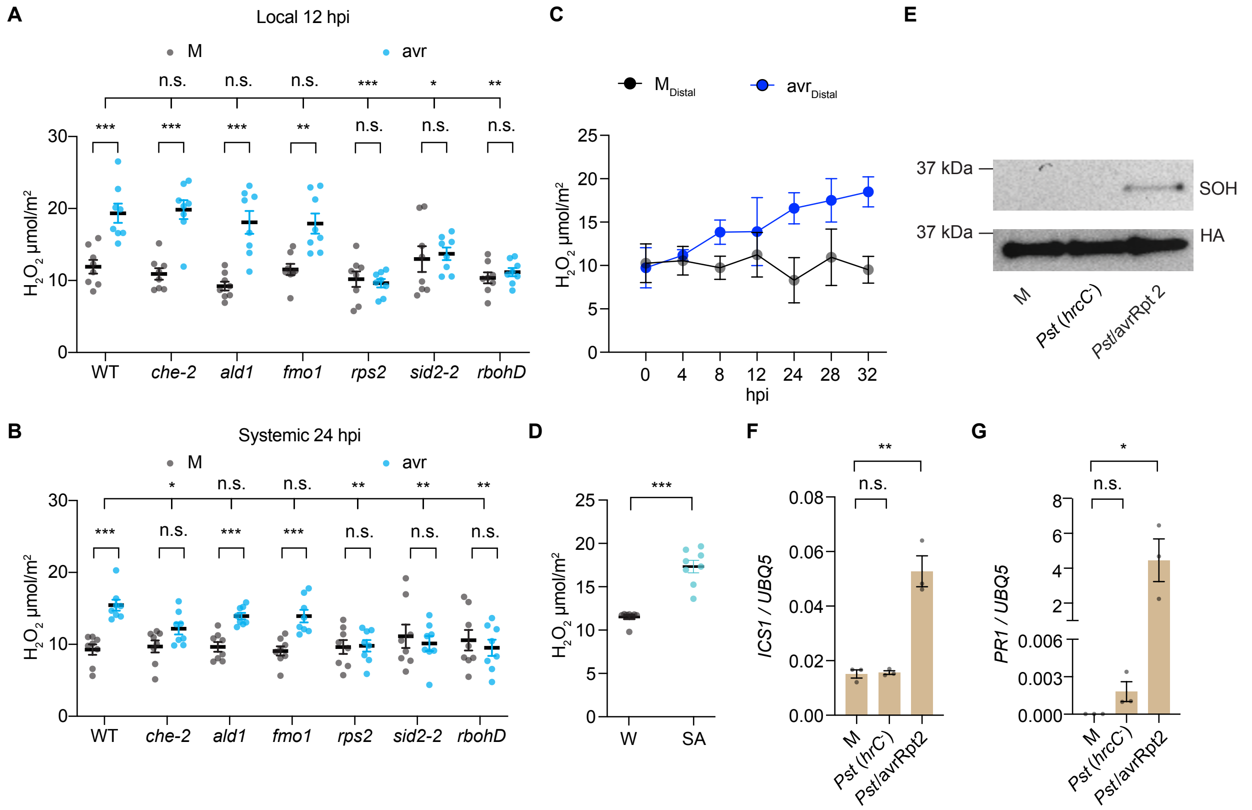

Fig. S7. H_2O_2 is the initial mobile signal for ETI-induced SAR. (**A** and **B**) Levels of H_2O_2 produced in the local tissues 12 hours after mock (M; 10 mM $MgCl_2$) or *Psm* ES4326/avrRpt2 (avr; OD_{600nm} = 0.01) treatment (A) or in the systemic tissues 24 hours after the local treatment (B). hpi, hours post infiltration. Data are means ± SEMs (*n* = 8). (**C**) Time-course H_2O_2 production in the distal tissues when lower leaves were treated with M or avr. Data are means ± SEMs (*n* = 5). (**D**) Levels of H_2O_2 produced 24 hours after water (W) or 1 mM SA treatment in WT plants. Data are the means ± SEMs (*n* = 8). (**E-G**) Sulfenylation of CHE (E) and expressions of *ICS1* (F) and *PR1* (G) in systemic tissues after local treatment with M, *Pst* DC3000 *$hrcC^-$* [*Pst* (*$hrcC^-$*)] or *Pst* DC3000/avrRpt2 (*Pst*/avrRpt2). Data are means ± SEMs (*n* = 3). Significant differences were calculated using either two-tailed Student's t-tests or two-way ANOVA. ***$P < 0.001$; **$P < 0.01$; *$P < 0.05$; n.s., not significant.

bioRxiv preprint doi: https://doi.org/10.1101/2023.07.27.550865; this version posted August 1, 2023. The copyright holder for this preprint (which was not certified by peer review) is the author/funder, who has granted bioRxiv a license to display the preprint in perpetuity. It is made available under aCC-BY 4.0 International license.

Fig S8

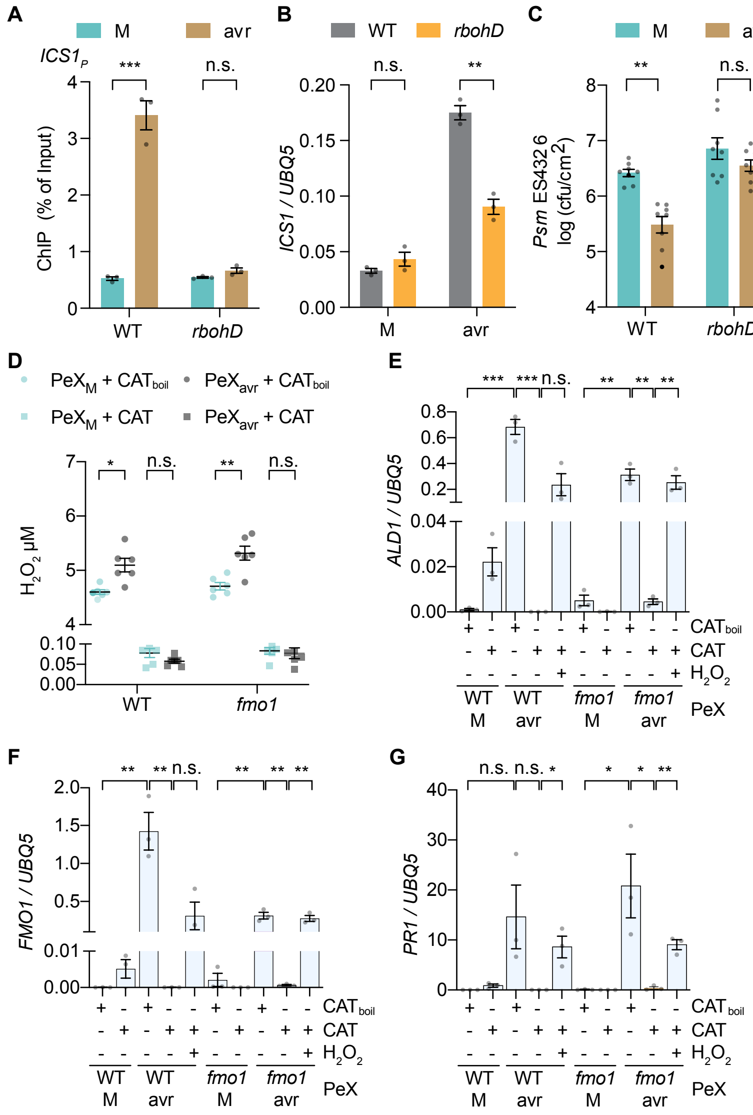

Fig. S8. H_2O_2 produced by RBOHD is the signal that induces systemic SA synthesis and Pip accumulation to confer SAR. (**A-C**) ChIP-qPCR analysis of CHE binding to the *ICS1* promoter carrying the TCP-binding site (*ICS1$_P$*) (A), transcriptional level of *ICS1* (B) and bacterial growth (C) in systemic tissues of WT or *rbohD* plants after local mock (M; 10 mM $MgCl_2$) or *Psm* ES4326/avrRpt2 (avr; OD_{600nm} = 0.01) treatment. Data are means ± SEMs. *n* = 3 for (A and B), *n* = 8 for (C). *ICS1$_P$*, the *ICS1* promoter sequence carrying the TCP-binding site. (**D**) Levels of H_2O_2 in petiole exudates (PeX) collected from WT and *fmo1* plants after treatment with catalase (CAT) or heat-denatured catalase (CAT_{boil}). Data are means ± SEMs (*n* = 6). (**E-G**) The expression of *ALD1* (E), *FMO1* (F) and *PR1* (G) after infiltration with PeX collected from WT or *fmo1* treated with M or avr. Data are means ± SEMs (*n* = 3). Significant differences were calculated using two-tailed Student's t-tests. ****P* < 0.001; ***P* < 0.01; **P* < 0.05; n.s., not significant.
